# Integrated Molecular Docking and Dynamic Simulations Reveal Glycyrrhizic Acid Alleviates Allergic Rhinitis in Rats by Inhibiting the TLR4/NF‐κB/IL‐1β Pathway

**DOI:** 10.1155/jimr/6032689

**Published:** 2026-08-10

**Authors:** Jianquan Huang, Xinyue Ren, Xiaojin Chen, Xiang Zheng

**Affiliations:** ^1^ Department of Pharmacy, Hangzhou Children’s Hospital, Hangzhou 310014, Zhejiang, China, hzch.org; ^2^ Department of Pharmacy, Hangzhou Maternity and Child Health Care Hospital, Hangzhou 310008, Zhejiang, China; ^3^ Pediatric Research Institute, Hangzhou Children’s Hospital, Hangzhou 310014, Zhejiang, China, hzch.org

**Keywords:** allergic rhinitis, glycyrrhizic acid, molecular docking, molecular dynamics simulation, TLR4/NF-κB/IL-1β pathway

## Abstract

**Background:**

Allergic rhinitis (AR) is a prevalent chronic inflammatory nasal disorder with suboptimal current therapies. Glycyrrhizic acid (GA), a bioactive triterpenoid from *Glycyrrhiza uralensis*, exerts well‐documented antiinflammatory effects, and preliminary evidence indicates its efficacy in alleviating airway inflammation in AR models. However, two critical knowledge gaps remain unaddressed: whether GA directly binds to core proteins of the toll‐like receptor 4 (TLR4)/nuclear factor‐kappa B (NF‐κB)/IL‐1β inflammatory pathway, and whether this binding mediates GA’s therapeutic effects in AR have not been systematically verified by integrated computational and in vivo experiments.

**Methods:**

An ovalbumin (OVA)‐induced AR rat model was established. GA was administered intranasally for seven consecutive days. Behavioral observations, histopathological examination, and serum cytokine detection were performed to evaluate AR‐related symptoms and immune imbalance. Molecular docking was employed to assess the binding affinity between GA and four key proteins in the TLR4 pathway (TLR4, myeloid differentiation primary response 88 [MyD88], NF‐κB, and IL‐1β). About 100 ns molecular dynamics (MD) simulations were further conducted to validate the stability of the GA–protein complexes. Immunohistochemistry and RT‐qPCR were used to verify the expression of pathway‐related proteins and cytokines in nasal mucosal tissues.

**Results:**

GA significantly reduced sneezing, rhinorrhea, and nasal mucosal pathological damage in AR rats. It restored the T helper 1 (Th1)/Th2 immune balance by suppressing Th2 cytokines (IL‐4 and IL‐13) and enhancing Th1 cytokines (interferon‐gamma [IFN‐γ] and IL‐2). Molecular docking results showed that GA bound strongly to all four target proteins, with the highest affinity for IL‐1β (−9.3 kcal/mol) and TLR4 (−7.6 kcal/mol). MD simulations confirmed the stable conformational dynamics of the GA–IL‐1β and GA–TLR4 complexes. In vivo experiments further demonstrated that GA significantly downregulated the expression of TLR4, MyD88, NF‐κB, and IL‐1β in nasal mucosa and reduced the levels of downstream proinflammatory cytokines (TNF‐α and IL‐6).

**Conclusions:**

GA alleviates AR symptoms by directly binding to core proteins of the TLR4/NF‐κB/IL‐1β pathway, inhibiting pathway activation, restoring Th1/Th2 immune balance, and suppressing inflammatory responses. This study provides structural and functional evidence supporting GA as a promising targeted therapeutic candidate for AR.

## 1. Introduction

Allergic rhinitis (AR) is a prevalent immunoglobulin E (IgE)‐mediated inflammatory disorder of the nasal mucosa, clinically manifested by sneezing, rhinorrhea, nasal congestion, and pruritus [1]. Affecting an estimated 10%–30% of the global population, AR substantially compromises quality of life, sleep quality, and cognitive performance [2]. The rising burden of environmental pollution is expected to further drive an increase in AR incidence worldwide [3, 4]. Despite the availability of current therapeutic modalities, including antihistamines, corticosteroids, and allergen‐specific immunotherapy, many patients experience inadequate long‐term symptoms control or suffer from treatment‐related adverse effects [5, 6]. These limitations underscore the pressing need for novel, safe, and efficacious therapeutic strategies.

AR is currently understood to arise from an immunological imbalance between T helper 1 (Th1) and Th2 lymphocytes, with a predominant skewing toward the Th2 phenotype [7]. The toll‐like receptor 4 (TLR4) signaling pathway plays a pivotal role in regulating Th2 immune responses and mediating allergic inflammation [8, 9]. Upon allergen exposure, TLR4 recognizes damage‐associated molecular patterns from inflamed epithelial cells, triggering myeloid differentiation primary response 88 (MyD88)‐dependent downstream signaling. This cascade ultimately activates nuclear factor‐kappa B (NF‐κB), which translocates to the nucleus and induces the expression of proinflammatory cytokines such as IL‐1β. These cytokines further promote Th2‐cell polarization and eosinophil recruitment in the nasal mucosa, sustaining chronic inflammation [10, 11]. Targeted inhibition of the TLR4/NF‐κB/IL‐1β axis is therefore a promising strategy for AR treatment.

Glycyrrhizic acid (GA) is a triterpenoid saponin derived from the roots of *Glycyrrhiza uralensis* Fisch. (Fabaceae), a widely used herb in traditional Chinese medicine. It possesses diverse pharmacological properties, including antiinflammatory, antioxidant, and antiapoptotic activities [12–14]. Given its demonstrated efficacy and favorable safety profile, GA has attracted growing interest as a potential therapeutic agent. Notably, prior studies have shown that GA alleviates airway inflammation in models of AR [15–17]. However, the precise molecular mechanisms underlying GA’s anti‐AR effects remain unclear. A critical knowledge gap exists: whether GA exerts its therapeutic effects by directly binding to core proteins of the TLR4/NF‐κB/IL‐1β pathway and disrupting their signaling function has not been systematically investigated.

The integration of computer‐aided drug design (including molecular docking and molecular dynamics [MD] simulations) with in vivo pharmacological validation provides a powerful approach to elucidate the molecular targets of natural compounds [18–21]. This strategy enables atomic‐level characterization of ligand–target interactions and dynamic assessment of binding stability, which can be further verified through in vivo experiments [22]. Against this backdrop, the present study aims to address two clearly defined scientific questions: Does GA directly bind to key proteins of the TLR4/NF‐κB/IL‐1β pathway with high affinity and stability? Does GA alleviate AR symptoms by inhibiting the TLR4/NF‐κB/IL‐1β signaling axis and restoring the Th1/Th2 immune balance? To answer these questions, we combined molecular docking and 100 ns MD simulations to analyze GA–protein interactions, followed by in vivo validation using an ovalbumin (OVA)‐induced AR rat model. This integrated approach provides structural and functional evidence for GA as a potential natural inhibitor of the TLR4 pathway, offering new insights into the development of targeted therapies for AR.

## 2. Materials and Methods

### 2.1. Materials and Reagent

GA was purchased from Shanghai Yuanye Biotechnology Co., Ltd. OVA was obtained from Sigma–Aldrich (United States of America). Aluminum hydroxide reagent was acquired from Macklin Biochemica Co., Ltd. H&E staining reagent was supplied by Shanghai Yuanye Biotechnology Co., Ltd. Rat ELISA kits for interferon‐gamma (IFN‐γ), IL‐2, IL‐4, and IL‐13 were procured from Wuhan Boster Biological Technology Co., Ltd. PBS buffer and DAB chromogen solution were provided by Servicebio Technology Co., Ltd. The SPARKscript II RT Plus Kit with the gDNA Eraser was purchased from Shandong Sikejie Biotechnology Co., Ltd. Primary antibodies against TLR4, MyD88, and NF‐κB were obtained from Wuhan Sanying Biotechnology Co., Ltd.

### 2.2. Animal Model Construction and Treatment

Twenty‐four male SPF‐grade SD rats aged 8 weeks with a body weight of 200 ± 10 g were obtained from Hangzhou Medical College. The animal production license number is SCXK(Zhe)2024‐0002, and the animal quality certificate number is 20250311Aazz01000180051. The experimental protocol was approved by the Experimental Animal Welfare Ethics Committee of the Zhejiang Academy of Traditional Chinese Medicine. After a 1‐week acclimatization period, the rats were randomly assigned into three groups based on body weight (eight animals per group) as follows: normal control group (NG), model control group (MG), and GA group. All groups except the NG underwent induction of an AR model. In the initial sensitization phase, rats received an intraperitoneal injection of a solution containing 0.3 mg of OVA and 30 mg of aluminum hydroxide adjuvant in 1 mL of 0.9% NaCl once weekly for three consecutive weeks. The booster sensitization phase began on day 22, on which a 2% OVA solution prepared in 0.9% NaCl was administered intranasally (50 μL per nostril) once daily for 7 consecutive days. Concurrently, from day 22 onward, the GA group received daily intranasal administration of 1% GA (50 μL per nostril), while the NG and MG groups received an equivalent volume of 0.9% NaCl solution.

### 2.3. Behavioral Observation

The rats were observed for sneezing, runny nose, and nasal rubbing within 30 min after 3 and 7 days of administration. Nasal allergic symptoms were evaluated using a cumulative scoring system. Nasal itching: Mild rubbing of the nose was assigned a score of 1; persistent or vigorous rubbing of the nasal area was assigned a score of 2. Sneezing: 1–3 sneezes received 1 point; 4–10 sneezes received 2 points; and more than 10 sneezes received 3 points. Rhinorrhea: Discharge reaching the anterior nares received 1 point; discharge extending beyond the anterior nares received 2 points; and profuse rhinorrhea covering the entire snout received 3 points. All behavioral scoring was performed by two independent researchers in a blinded manner.

### 2.4. Detection of Serum Th1 and Th2 Cytokines

Blood was collected from anesthetized rats via the abdominal aorta. The samples were centrifuged at 3000 rpm for 15 min, and the resulting serum supernatants were aliquoted and stored at −80°C until further analysis. Prior to the assay, serum samples were thawed on ice. Concentrations of Th1 cytokines (IFN‐γ and IL‐2) and Th2 cytokines (IL‐4 and IL‐13) were determined using commercial ELISA kits, strictly following the manufacturer’s protocols. A standard curve was generated in each plate to enable the accurate quantification of cytokine levels in the serum samples.

### 2.5. Histopathological Examination

Following blood collection, rats were euthanized by cervical dislocation. The skin over the head and face was carefully removed, and the mandible was excised to facilitate access to the nasal cavity. The skull was bisected along the midline suture of the nasal bones, extending from the nasal tip toward the cranium. The entire nasal septum was then carefully dissected out, and the posterior one‐third adjacent to the cranial cavity was removed to minimize contamination from nonnasal tissues. Nasal mucosal specimens were collected, fixed in 4% paraformaldehyde, embedded in paraffin, and sectioned. The sections were deparaffinized, rehydrated through a graded ethanol series, stained with H&E, dehydrated, and examined under light microscopy to assess the histopathological changes in the nasal mucosa.

The blinded semiquantitative scoring system was adopted to evaluate epithelial injury, inflammatory infiltration, and mucosal edema in H&E‐stained nasal mucosal sections. Specifically, 0 point was assigned for intact mucosal epithelial structure without edema and inflammatory cell infiltration; 1 point for basically intact mucosal epithelial structure with only minimal cellular edema, no obvious tissue edema, and a small number of scattered inflammatory cells; 2 points for mild mucosal epithelial injury, disordered local cell arrangement, slight edema in the superficial mucosa, and mild inflammatory cell infiltration; 3 points for moderate damage and partial exfoliation of mucosal epithelium, obvious edema and widened interstitial spaces in the lamina propria, as well as significantly increased inflammatory cell infiltration; 4 points for extensive exfoliation and severely impaired structural integrity of mucosal epithelium, severe mucosal edema and loosening, and massive aggregated infiltration of inflammatory cells.

### 2.6. Molecular Docking Analysis

Molecular docking was performed between GA and the proteins encoded by TLR4, MyD88, NF‐κB, and IL‐1β to preliminarily assess their potential interactions. The three‐dimensional protein structures corresponding to these four targets were retrieved from the UniProt Knowledgebase (https://www.uniprot.org/uniprotkb) and downloaded in the PDB format [23]. The chemical structure of GA was obtained from the PubChem database (https://pubchem.ncbi.nlm.nih.gov) [24]. Molecular docking simulations between GA and each target protein were carried out using AutoDock Vina [25]. Binding affinities were recorded, and the resulting protein–ligand complexes were visualized using Discovery Studio software [26].

### 2.7. MD Simulations

MD simulations were performed to further validate the interactions between the target proteins and GA over a 100 ns timescale. Based on the molecular docking results, two protein–ligand complexes, GA bound to IL‐1β and GA bound to TLR4, were selected for subsequent analysis. A 100 ns MD simulation was carried out using GROMACS 2024.2 to evaluate the plausibility and reliability of the docking poses [27]. The AMBER/GAFF force field parameters for the ligand were generated using the Sobtop program (Tian Lu, Sobtop, Version 1.0 [dev5], http://sobereva.com/soft/Sobtop) [28]. Protein parameters and topologies were assigned using the built‐in AMBER14SB force field in GROMACS 2024.2. Periodic boundary conditions were set and optimized to define the size of the simulation box, which was then filled with water molecules. To ensure the overall electroneutrality of the simulation system, a portion of the solvent water molecules was replaced with Na^+^ and Cl^−^ ions at a concentration of 0.15 mol/L. The steepest descent method was employed to minimize the total potential energy of the system. The equilibration procedure was carried out in two sequential stages. In the first stage, an NVT ensemble simulation was performed at 300 K for 100 ps to stabilize the system temperature. In the second stage, an NPT ensemble simulation was conducted at 1 bar for 100 ps to equilibrate and stabilize the system pressure. The resulting trajectories were visualized using DuIvyTools (https://duivytools.readthedocs.io/en/v0.6.0/DIT.html) [29].

### 2.8. Detection of TLR4, MyD88, and NF‐κB Expression in Nasal Mucosa by Immunohistochemistry

Nasal mucosal tissues were embedded in paraffin and sectioned at a thickness of 5 μm. The sections underwent sequential deparaffinization, antigen retrieval, and blocking. Following rinsing with PBS, primary antibodies against TLR4, MyD88, and NF‐κB diluted 1–200 were applied and incubated overnight at 4°C in a humidified chamber. On the following day, the sections were incubated with the HRP‐conjugated secondary antibody at room temperature for 30 min. After chromogenic detection with DAB, the sections were counterstained, dehydrated, and coverslipped. Immunostaining was visualized under a light microscope, and protein expression levels were semiquantitatively assessed using ImageJ software. The integrated optical density (IntD) within the selected region was calculated and expressed as the mean optical density per unit area. The ImageJ semiquantitative analysis of IntD was conducted under blinded conditions to avoid subjective bias.

### 2.9. Detection of TLR4, MyD88, NF‐κB, IL‐1β, IL‐6, and TNF‐α Expression in Nasal Mucosa by RT‐qPCR

Nasal mucosal tissues were collected from rats, and total RNA was isolated using a rapid total RNA extraction kit. The concentration and purity of the extracted RNA were determined spectrophotometrically. Reverse transcription was carried out in accordance with the protocol provided by the manufacturer for the SPARKscript II RT Plus Kit with the gDNA Eraser. RNA was combined with the gDNA removal solution and RNase‐free ddH_2_O, and the mixture was incubated at 42°C for 2 min. Stem‐loop primers, RT premix, and reverse transcriptase were then added, and the reverse transcription reaction was carried out in a thermal cycler. Following reverse transcription, gene‐specific primers were designed, and quantitative amplification was performed using an RT‐qPCR system (Table 1). Relative mRNA expression levels were calculated using the 2^−ΔΔCt^ method. β‐Actin was used as the housekeeping (reference) gene for normalization due to its stable constitutive expression in rat nasal mucosal tissues.

**Table 1:** The sequences of primers.

Genes	Sequences(5′–3′)	Sequences(3′–5′)
TLR4	TGTATCGGTGGTCAGTGTGC	CAGCTCGTTTCTCACCCAGT
MyD88	GAGAGCAGTGTCCCACAGAC	AGCAGATGAAGGCGTCGAAA
NF‐κB	CAGACACCTTTGCACTTGGC	CTTGAGTAGGACCCCGAGGA
IL‐1β	CACCTCTCAAGCAGAGCACA	CGGGTTCCATGGTGAAGTCA
IL‐6	AGAGACTTCCAGCCAGTTGC	AGCCTCCGACTTGTGAAGTG
TNF‐α	CTGTGCCTCAGCCTCTTCTC	ACTGATGAGAGGGAGCCCAT
β‐Actin	CCCGCGAGTACAACCTTCT	CGTCATCCATGGCGAACT

*Note:* β‐Actin serves as the housekeeping gene for RT‐qPCR normalization.

### 2.10. Statistical Analysis

The data are presented as the mean ± standard deviation (SD). One‐way analysis of variance (ANOVA) was used to evaluate statistical differences among groups, followed by Tukey’s multiple comparison test for posthoc intergroup comparisons. Differences with *p* < 0.05 were considered statistically significant. All quantitative data plotting was performed using Origin 8.5 software.

A total of 24 SPF‐grade SD rats were randomly divided into three groups with 8 animals per group (*N* = 8). This sample size was determined based on preexperimental data, published literature on OVA‐induced AR rat models, and laboratory animal 3Rs ethical principles. Posthoc statistical power analysis was performed using the main quantitative indicators in this study. The statistical power of all primary outcome measures was higher than 0.8, demonstrating that the current sample size has adequate statistical efficacy to distinguish significant differences among groups. A priori power analysis was performed in our subsequent confirmatory experiments to optimize the sample size in advance.

## 3. Results

### 3.1. GA Alleviates Nasal Symptoms in OVA‐Induced AR Rats

**Figure 1:**
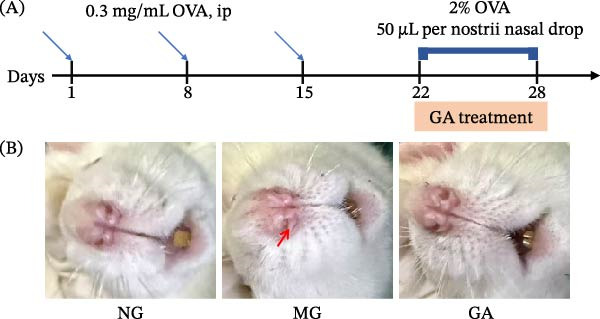
Establishment of an OVA‐induced allergic rhinitis model and general nasal manifestations of rats. (A) Models of OVA‐induced AR and pharmacologic treatment protocol. (B) Representative images of nasal symptoms in rats from the normal control group (NG), model control group (MG), and GA treatment group. The red arrow indicates a runny nose. Eight rats were included in each group (*n* = 8).

AR was induced in rats by intraperitoneal sensitization with OVA formulated with an aluminum hydroxide adjuvant, followed by repeated intranasal OVA challenges (Figure 1). The model rats exhibited symptoms consistent with those observed in humans, including frequent sneezing, vigorous nasal rubbing, and clear rhinorrhea. Administration of GA significantly attenuated these manifestations, as evidenced by a marked reduction in both nasal rubbing frequency and sneeze counts (Table 2). Furthermore, the total nasal symptoms score in the GA‐treated group was significantly lower than that in the MG group (*p* < 0.05, 0.01).

**Table 2:** Effect of GA on nasal symptoms in AR rats.

Group	Nasal symptoms scores after drug administration	
	Score on day 3	Score on day 7
NG	0.3 ± 0.5	1.0 ± 0.7
MG	3.7 ± 0.9^##^	5.7 ± 0.9^##^
GA	2.8 ± 0.8^∗^	4.2 ± 1.2^∗∗^

*Note:* Data are presented as mean ± standard deviation (SD). Each group contains 8 rats (*n* = 8). All assays were repeated three times independently. Statistical analysis was performed using one‐way ANOVA with Tukey’s multiple comparison test.

^##^ *p* < 0.01 vs. NG.

^∗^ *p* < 0.05.

^∗∗^ *p* < 0.01 vs. MG.

### 3.2. GA Regulates Serum Th1/Th2 Cytokines and Attenuates Histopathological Damage in the Nasal Mucosa

**Figure 2:**
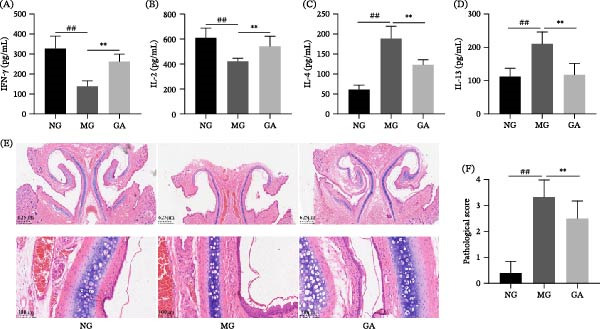
GA regulates Th1/Th2 cytokine balance and alleviates nasal mucosal pathological damage in AR rats. (A–D) Serum levels of IFN‐γ, IL‐2, IL‐4, and IL‐13 detected by ELISA. (E) Representative H&E staining images of rat nasal mucosa (scale bar as indicated). (F) Pathological score. Data are presented as mean ± standard deviation (SD). Each group contains 8 rats (*n* = 8). All assays were repeated 3 times independently. Statistical analysis was performed using one‐way ANOVA with Tukey’s multiple comparison test. ^##^ *p* < 0.01 vs. NG;  ^∗∗^ *p* < 0.01 vs. MG.

Th1/Th2 cell‐mediated immune imbalance represents a central mechanism underlying the AR. The function of Th1 cells is the secretion of cytokines, such as IL‐2 and IFN‐γ, which are pivotal in the regulation of cellular immune responses. In contrast, Th2 cells are responsible for the production of cytokines, including IL‐4 and IL‐13, which are essential in the promotion of humoral immunity. IL‐4 and IFN‐γ are frequently used as biomarkers to assess the polarization of the Th subset. IFN‐γ plays a pivotal role in suppressing Th2‐driven inflammation in the nasal mucosa by inhibiting mast cell‐mediated IgE production and reducing IL‐4 secretion. Conversely, IL‐4 enhances IgE synthesis and facilitates eosinophil recruitment into the nasal mucosa, thereby amplifying the inflammatory response. In this study (Figure 2A–D), AR model rats exhibited significantly elevated serum levels of Th2 cytokines IL‐4 and IL‐13, accompanied by a marked reduction in Th1 cytokines IL‐2 and IFN‐γ. Notably, GA administration effectively reversed this imbalance, resulting in a significant decrease in IL‐4 and IL‐13 levels and a concomitant increase in IL‐2 and IFN‐γ concentrations (*p* < 0.01).

H&E staining showed that the nasal mucosa of rats in the NG exhibited intact and well‐organized histological architecture with no evidence of edema or vascular congestion. The epithelial layer and the underlying lamina propria were clearly delineated. In contrast, a marked pathological change was observed in MG. The lamina propria exhibited marked edema and vascular congestion, accompanied by submucosal glandular hyperplasia, neovascularization, and extensive infiltration of inflammatory cells, predominantly eosinophils. Furthermore, the ciliated epithelium on the mucosal surface was severely disrupted. Notably, the nasal mucosal structure was largely preserved in the GA‐treated group. Only sparse eosinophil infiltration was detected, and signs of congestion, edema, and glandular hyperplasia were substantially attenuated (Figure 2E). Consistent with morphological observations, histological scoring results showed that the scores of mucosal edema, epithelial damage, and eosinophil infiltration were significantly increased in the model group compared with the normal group (*p* < 0.01). After GA intervention, all pathological scores were significantly decreased (*p* < 0.01), further confirming that GA alleviated nasal mucosal lesions in AR rats (Figure 2F).

### 3.3. Molecular Docking of GA With Key Target Proteins

**Figure 3:**
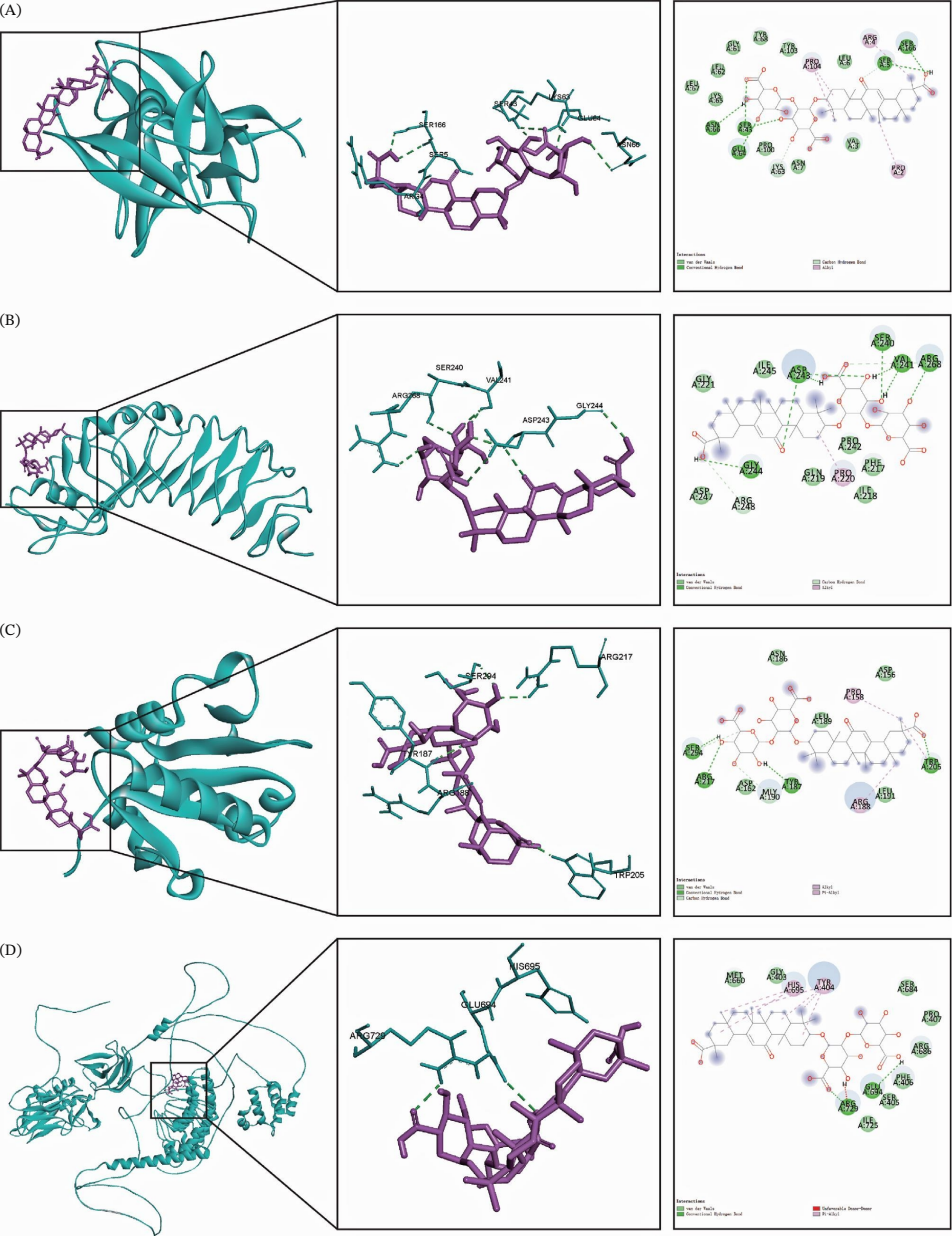
Molecular docking analysis between GA and four key proteins of the TLR4/NF‐κB/IL‐1β pathway. (A) GA–IL‐1β complex; (B) GA–TLR4 complex; (C) GA–MyD88 complex; and (D) GA–NF‐κB complex. Overall structure, a local enlarged view, and 2D interaction diagrams are displayed for each complex.

As described in the Methods section, molecular docking was performed between GA and the proteins encoded by IL‐1β, TLR4, MyD88, and NF‐κB. The three‐dimensional structures of these target proteins were retrieved from the UniProt database, and the chemical structure of GA was downloaded from PubChem. Molecular docking simulations between GA and each of the four proteins were carried out using AutoDock Vina, and the corresponding binding free energies were evaluated. A more negative binding free energy indicates stronger binding affinity, and interactions with a binding free energy ≤ −5.0 kcal/mol are generally considered to represent stable and specific binding. The results showed that GA exhibited strong binding affinities toward all four target proteins. Moreover, GA formed multiple conventional hydrogen bonds with both IL‐1β and TLR4 (Table 3). Subsequently, the protein–ligand complexes were visualized using Discovery Studio software to illustrate the binding modes and key interactions (Figure 3).

**Table 3:** Molecular docking binding energy and hydrogen bond number.

Ligand–receptor relevant information	Protein‐PDB	Affinity (kcal/mol)	Conventional hydrogen bond	Carbon hydrogen bond
GA–IL‐1β	3POK	−9.3	6	2
GA–TLR4	2Z62	−7.6	8	2
GA–MyD88	4DOM	−7.8	4	2
GA–NF‐κB	AF‐P19838‐F1	−7.5	2	0

### 3.4. MD Simulations of GA With Core Target Proteins

**Figure 4:**
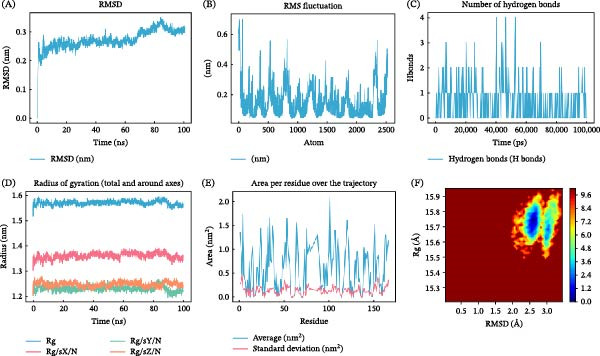
Dynamic characteristics of the GA–IL‐1β complex during 100 ns molecular dynamics simulation. (A) Root mean square deviation. (B) Root mean square fluctuation. (C) Number of hydrogen bonds. (D) Radius of gyration. (E) Solvent‐accessible surface area. (F) Free energy landscape maps.

**Figure 5:**
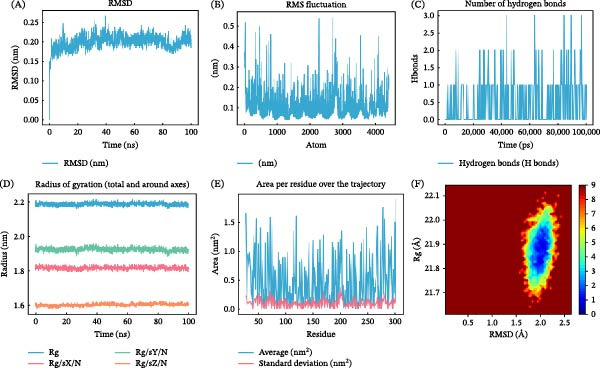
Dynamic characteristics of the GA–TLR4 complex during 100 ns molecular dynamics simulation. (A) Root mean square deviation. (B) Root mean square fluctuation. (C) Number of hydrogen bonds. (D) Radius of gyration. (E) Solvent‐accessible surface area. (F) Free energy landscape maps.

Two protein–ligand complexes, GA–IL‐1β and GA–TLR4, were selected from the molecular docking results for further validation of binding stability through 100 ns MD simulations. The root mean square deviation (RMSD) was employed to evaluate conformational stability. RMSD quantifies the average displacement of corresponding atoms between a reference structure, typically the initial frame, and subsequent frames, thereby reflecting structural drift during the simulation. The RMSD trajectories of both the protein and the ligand are widely used indicators to assess whether the system has reached dynamic equilibrium. The results showed that the IL‐1β protein in the GA–IL‐1β complex exhibited an RMSD plateauing around 0.3 nm, indicating that the protein conformation stabilized during the 70–100 ns interval (Figure 4A). In contrast, the TLR4 protein in the GA–TLR4 complex displayed RMSD values consistently within the range of 0.15–0.25 nm throughout the entire 100 ns simulation, suggesting immediate and sustained structural stability from 0–100 ns (Figure 5A). Collectively, these RMSD profiles confirm that both GA–IL‐1β and GA–TLR4 complexes maintain stable conformations during the simulation, thereby supporting the reliability and plausibility of the predicted binding poses.

The root mean square fluctuation (RMSF) represents the time‐averaged deviation of atomic positions and is used to characterize the flexibility and dynamic behavior of individual amino acid residues throughout the simulation. Analysis of the RMSF profiles indicates that both systems achieved equilibrium, with no signs of global anomalous fluctuations, confirming the structural integrity of the proteins over the entire simulation period (Figures 4B and 5B). Furthermore, the plot of the number of hydrogen bonds demonstrates that hydrogen‐bonding interactions underwent continuous and rapid formation and dissociation during the simulation. No persistent upward or downward trend was observed, suggesting a dynamic yet stable interaction pattern between the ligand and the protein (Figures 4C and 5C).

The radius of gyration (Rg) is a key metric for assessing the overall compactness of protein‐small molecule complexes and can be used to characterize changes in the looseness or tightness of the protein backbone during the simulation. As illustrated in the Rg profiles, both the IL‐1β‐GA and TLR4‐GA systems remained structurally stable throughout the 100 ns simulation period, without evidence of phase transitions, and displayed marked anisotropy (Figures 4D and 5D). Solvent‐accessible surface area (SASA) analysis provides insights into the degree of ligand exposure to the surrounding solvent and indirectly reflects how tightly the protein envelops the ligand. As demonstrated in the SASA profiles, the SASAs of individual residues remained relatively stable across the entire trajectory, indicating that both systems achieved convergence (Figures 4E and 5E).

The free‐energy landscape maps the conformational space of the protein to its free‐energy levels, with colors ranging from red to blue representing high to low free energy. Bluer colors indicate lower‐energy states and greater conformational stability. In the free‐energy distribution plot for the GA–IL‐1β complex, two energy wells were identified, indicating that the GA–IL‐1β complex primarily resides in two distinct conformational subspaces. The primary energy well, located at RMSD ~2.6 Å and Rg ~15.75 Å, shows the deepest blue color, suggesting that this conformation is the most stable. The secondary energy well at RMSD ~3.1 Å and Rg ~15.65 Å displays a slightly lighter blue color, indicating that it is also stable but with marginally higher free energy, thus representing a metastable or intermediate conformation (Figure 4F). For the GA–TLR4 complex, the free‐energy distribution plot revealed a single energy well, indicating that the GA–TLR4 complex mainly exists within a single conformational subspace. The energy minimum point is located near RMSD ~2.0 Å and Rg ~21.9 Å, showing a deep blue color, which signifies the lowest free energy and highest stability (Figure 5F). These results further confirm the targeted binding capability between GA and both IL‐1β and TLR4.

### 3.5. GA Alleviates AR by Inhibiting the TLR4/NF‐κB/IL‐1β Pathway

**Figure 6:**
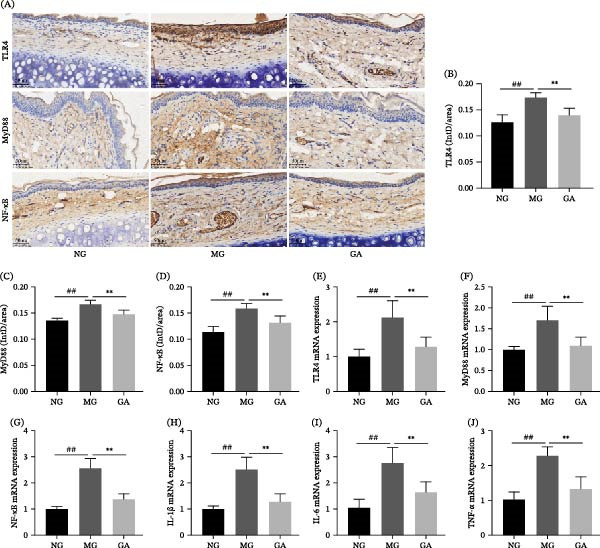
GA inhibits the activation of the TLR4/NF‐κB/IL‐1β signaling pathway in rat nasal mucosa. (A) Representative immunohistochemical staining of TLR4, MyD88 and NF‐κB. (B) The integrated optical density of TLR4. (C) The integrated optical density of MyD88. (D) The integrated optical density of NF‐κB. (E–J) Relative mRNA expression of TLR4, MyD88, NF‐κB, IL‐1β, IL‐6 and TNF‐α detected by RT‐qPCR. Data are presented as mean ± standard deviation (SD). Each group contains 8 rats (*n* = 8). All assays were repeated 3 times independently. Statistical analysis was performed using one‐way ANOVA with Tukey’s multiple comparison test. ^##^ *p* < 0.01 vs. NG;  ^∗∗^ *p* < 0.01 vs. MG.

The TLR4/NF‐κB/IL‐1β signaling pathway is a central regulator of inflammatory responses and plays a dominant role in the pathogenesis of AR. Immunohistochemical analysis showed that treatment with GA significantly downregulated the protein expression of TLR4, MyD88, and NF‐κB in the nasal mucosa of AR model rats (*p* < 0.01). Furthermore, the results of RT‐qPCR demonstrated that the mRNA expression of TLR4, MyD88, NF‐κB, IL‐1β, IL‐6, and TNF‐α was significantly increased in the nasal mucosa of model rats in comparison with normal controls. The upregulation was effectively reversed by GA administration (*p* < 0.01). Collectively, these results suggest that GA ameliorates AR by suppressing the activation of the TLR4/NF‐κB/IL‐1β signaling axis and attenuating the downstream inflammatory cascade (Figure 6).

## 4. Discussion

AR is a chronic inflammatory disorder of the nasal mucosa primarily induced by exposure to specific allergens in sensitized individuals. Although not life‐threatening, AR is notoriously difficult to cure and prone to frequent relapse. Its hallmark clinical features include intermittent or persistent nasal congestion, sneezing, and watery rhinorrhea, all of which considerably disrupt daily activities and diminish the quality of life [30]. A well‐established approach for modeling AR in rodents involves systemic sensitization via intraperitoneal injection of OVA, followed by local intranasal challenge [31]. In this study, an AR rat model was generated using the following protocol. Behavioral assessment showed that OVA‐sensitized and ‐challenged rats developed evident symptoms, including frequent sneezing and rhinorrhea, confirming successful induction of the disease phenotype. Administration of GA significantly reduced the frequency of sneezing and nose‐rubbing episodes, suggesting that GA effectively ameliorates clinical manifestations in AR rats.

An imbalance between Th1 and Th2 cytokines has been demonstrated to drive excessive infiltration of eosinophils and mast cells into the nasal mucosa, leading to local inflammation and tissue injury [32]. This has been identified as a key pathogenic mechanism in AR. In this study, rats in the model group exhibited pronounced structural disruption of the nasal mucosa, accompanied by extensive eosinophil infiltration. These pathological changes were markedly ameliorated following treatment with GA. Eosinophil recruitment is tightly linked to aberrant Th2‐cell activation. Upon activation, Th2 cells proliferate and secrete IL‐4 and IL‐13, which promote the migration and accumulation of eosinophils in the nasal mucosa, thereby initiating and sustaining inflammation [33, 34]. Concurrently, Th2 hyperactivity suppresses Th1 responses, resulting in reduced frequencies of Th1 cells and decreased production of their signature cytokines, IFN‐γ and IL‐2. The function of macrophages is modulated by IFN‐γ, while IL‐2 enhances the proliferation and activation of natural killer cells, which in turn leads to the suppression of IgE synthesis [35]. It is noteworthy that the intervention of GA led to a substantial reduction in serum levels of IL‐4 and IL‐13 while concurrently resulting in a marked elevation in those of IL‐2 and IFN‐γ in AR model rats. These findings underscore the capacity of GA to restore Th1/Th2 homeostasis and mitigate allergic inflammation in AR.

Modern medical research has established that inflammatory responses underlie the entire pathogenesis of AR from onset through progression [36]. Consequently, investigations centered on inflammation‐driven pathological mechanisms are pivotal for identifying novel therapeutic targets in AR. The TLR4/NF‐κB/IL‐1β signaling pathway serves as a central regulatory node in the inflammatory cascade and is intimately linked to AR development through the orchestration of proinflammatory responses [10]. Nevertheless, whether GA exerts protective effects against AR by modulating this signaling axis remains unknown. Advances in structural biology have enabled the application of computational approaches such as molecular docking and MD simulations, which offer efficient, precise, and economical strategies for the virtual screening of drug target interactions. To address the core scientific question of this study, we employed in silico modeling to systematically evaluate the binding potential of GA against key inflammatory proteins IL‐1β, TLR4, MyD88, and NF‐κB. Furthermore, 100 ns MD simulations were performed to characterize the dynamic stability and interaction profiles of GA with IL‐1β and TLR4, thereby providing insights into the mechanistic basis of GA’s antiinflammatory and anti‐AR effects.

Molecular docking is a computational approach that simulates the molecular recognition process between ligands and target proteins and has become a cornerstone in drug design, virtual screening, and mechanistic pharmacological studies [37]. AutoDock is a widely used molecular docking program that employs a genetic algorithm to explore conformational space and has been extensively applied in drug discovery based on the molecular structure. In the present study, the binding free energies between GA and the four inflammatory targets IL‐1β, TLR4, MyD88, and NF‐κB were all below −5 kcal/mol, indicating strong and stable interactions. This result provides direct computational evidence that GA can bind to core proteins of the TLR4/NF‐κB/IL‐1β pathway with high affinity, which answers the first scientific question at the molecular level. Despite its utility, molecular docking has inherent limitations, as it typically treats biomolecules as rigid entities and fails to account for the dynamic complexity of protein–ligand interactions in solution.

To overcome these constraints, MD simulations have emerged as a powerful complementary strategy capable of capturing the time‐resolved conformational behavior of biomolecular complexes [38]. Such simulations not only validate docking predictions but also enhance the reliability of virtual screening by revealing the dynamic stability and interaction networks at an atomic resolution [39, 40]. Accordingly, this study employed GROMACS, a robust and widely validated platform for biomolecular simulations. Given that the accuracy of MD critically depends on the underlying force field, which provides a simplified yet physically meaningful representation of molecular energetics, the AMBER14SB force field was selected for its well‐established performance in modeling protein–ligand systems [41]. This force field was used to simulate the complexes of GA with the two core targets, IL‐1β and TLR4, to ensure high fidelity in structural and energetic characterizations. Through integrated analyses of RMSD, RMSF, Rg, SASA, and hydrogen bond dynamics, the simulations demonstrated that GA forms thermodynamically stable and structurally coherent complexes with both IL‐1β and TLR4. These results provide compelling computational evidence that IL‐1β and TLR4 are plausible molecular targets mediating the anti‐allergic effects of GA in AR. These results further confirm the targeted binding capability between GA and both IL‐1β and TLR4 and solidify the answer to the first scientific question from the perspective of dynamic molecular interactions.

TLR4 was the first TLR to be identified and is abundantly expressed on dendritic cells, macrophages, and mucosal epithelial cells in the nasal mucosa [42]. Upon recognition of its ligands, TLR4 initiates intracellular signaling cascades that drive the transcription of numerous genes, resulting in the enhanced production of inflammatory mediators and subsequent activation of immune responses. MyD88 serves as a pivotal adaptor protein whose C‐terminal domain interacts with the TIR domain of activated TLR4 to form a signaling complex [43]. This assembly triggers the downstream activation of NF‐κB. Under basal conditions, NF‐κB remains sequestered in the cytoplasm in an inactive state. Upon stimulation, the p65 subunit of NF‐κB translocates into the nucleus, where it promotes the expression of a wide spectrum of proinflammatory cytokines, thereby amplifying the inflammatory cascade [44]. During pathogen challenge, TLR4 signaling is engaged through both MyD88‐dependent and ‐independent pathways. The MyD88‐dependent route ultimately leads to nuclear translocation of NF‐κB and robust induction of inflammatory mediators, including IL‐1β, TNF‐α, and IL‐6, all of which are critically involved in the pathogenesis of AR [45]. Our results showed that the expression levels of TLR4, MyD88, NF‐κB and IL‐1β in the nasal mucosa of AR rats were significantly elevated compared with those in normal rats, indicating activation of the TLR4/NF‐κB/IL‐1β signaling axis in AR. Following the intervention of GA, the expression of TLR4, MyD88, NF‐κB, and IL‐1β in the nasal mucosa was markedly reduced, accompanied by a significant decrease in the release of proinflammatory cytokines TNF‐α and IL‐6. These results strongly suggest that GA alleviates AR by inhibiting the TLR4/NF‐κB/IL‐1β signaling pathway, thereby attenuating the downstream inflammatory response. These in vivo experimental data directly link the molecular binding capacity of GA to the inhibition of pathway activity and, together with the improvement of AR symptoms and Th1/Th2 balance, provide a complete answer to the two core scientific questions of this study.

It is necessary to distinguish the pathway‐specific inhibitory effect of GA from its general antiinflammatory activity. Three lines of evidence support that GA exerts anti‐AR efficacy mainly via specific inhibition of the TLR4/NF‐κB/IL‐1β pathway. First, molecular docking and MD simulations demonstrated that GA could directly bind to TLR4, MyD88, NF‐κB, and IL‐1β with high affinity and stable conformation, which is the essential evidence for targeted interaction. Second, in vivo results revealed an orderly cascade downregulation of all molecules along the TLR4/NF‐κB/IL‐1β signaling axis, showing typical characteristics of pathway‐specific regulation rather than nonspecific inhibition of scattered inflammatory factors. Third, the decreased activity of this pathway was highly consistent with the restoration of the Th1/Th2 immune balance and the alleviation of nasal mucosal lesions. Although GA has been reported to have broad‐spectrum antiinflammatory properties, the above evidence indicates that the therapeutic mechanism of GA against AR is dominated by targeted inhibition of the TLR4/NF‐κB/IL‐1β pathway. In further research, TLR4 agonist rescue experiments will be performed to more rigorously verify the pathway specificity.

The OVA‐induced acute rat AR model has certain translational limitations: it fails to simulate the chronic recurrent features, multiallergen stimulation, and persistent mucosal remodeling of human AR. Species differences in nasal structure and immune regulation also restrict direct data translation. However, the TLR4/NF‐κB/IL‐1β pathway and Th1/Th2 imbalance are conserved pathogenic mechanisms across species, so our findings on GA’s anti‐allergic and antiinflammatory effects have good clinical applicability. The intranasal administration route is also clinically feasible. For further clinical transformation, we need to evaluate the long‐term safety of GA, standardize its preparation, and establish more clinically relevant chronic AR models. Preliminary clinical trials will be carried out in the follow‐up work.

This work is an exploratory preclinical study to reveal the specific binding and anti‐allergic mechanism of GA targeting the TLR4/NF‐κB/IL‐1β axis, and the core conclusions are sufficiently supported by integrated computational simulation and in vivo multi‐index data. There are several minor limitations constrained by experimental conditions and research stage, which we acknowledge as follows: First, we adopted a single intranasal concentration of GA for intervention to prioritize the verification of core molecular interactions. Subsequent gradient dose experiments were carried out to further define the optimal therapeutic window. Besides, standard anti‐allergic positive drugs were not set in this mechanistic research; comparative efficacy evaluation will be completed in our follow‐up pharmacodynamic study. Second, the immune and pathological evaluation systems can be further supplemented. We detected representative Th1/Th2 cytokines and observed eosinophil infiltration via H&E staining, while classic allergic markers such as total IgE were not measured at this stage. Nasal mucosal lesions were analyzed descriptively, and standardized semiquantitative scoring will be supplemented in our next experiment. Third, tissue sample volume limited additional multilevel protein detection. Immunohistochemistry and serum ELISA combined with RT‐qPCR have confirmed the regulatory effect of GA on pathway molecules, while Western blot and tissue homogenate ELISA will be added in future work to strengthen transcriptional and translational consistency verification. Fourth, the computational simulation part can be further optimized. We validated the stable binding of GA–TLR4 and GA–IL‐1β through comprehensive 100 ns MD multidimensional analysis. Replicate MD trajectories and MM‐PBSA/MM‐GBSA thermodynamic calculations were supplemented to provide more abundant binding evidence. Although the above extensions can enrich our findings, the multidimensional evidence obtained in the current work reliably supports our main conclusion that GA alleviates AR via targeted inhibition of the TLR4/NF‐κB/IL‐1β pathway. We will systematically carry out the above supplementary experiments in follow‐up research to deepen and expand this research system.

## 5. Conclusion

This study systematically addressed two core scientific questions via an integrated approach combining molecular docking, MD simulations, and in vivo animal experiments. First, computational assays provided direct structural evidence that GA binds with high affinity to the core proteins of the TLR4/NF‐κB/IL‐1β pathway, forming stable molecular complexes. Among these interactions, the binding of GA to IL‐1β and TLR4 exhibited the strongest affinity, confirming these two proteins as key molecular targets of GA. Second, in vivo experiments validated the functional relevance of these binding events in OVA‐induced AR rats: GA administration significantly alleviated nasal symptoms including sneezing and rhinorrhea, ameliorated nasal mucosal pathological damage, and restored the Th1/Th2 immune balance by upregulating Th1 cytokines (IFN‐γ, IL‐2) and downregulating Th2 cytokines (IL‐4 and IL‐13). Concurrently, GA suppressed the expression of proinflammatory mediators (IL‐6 and TNF‐α) by inhibiting the activation of the TLR4/NF‐κB/IL‐1β signaling axis in nasal mucosal tissues. Collectively, these findings reveal a clear mechanistic link between GA’s molecular binding capacity and its anti‐AR efficacy: GA exerts therapeutic effects by targeting the TLR4/NF‐κB/IL‐1β pathway to suppress inflammatory responses and restore immune homeostasis. This study provides both structural and functional evidence supporting GA as a promising targeted therapeutic candidate for AR treatment.

## Author Contributions

Jianquan Huang contributed to the study conception and design. Xinyue Ren performed the experiments and analyzed the data. Xiaojin Chen edited and reviewed the manuscript. Xiang Zheng was responsible for the investigation, methodology, and original draft writing.

## Funding

This work was supported and sponsored by the Hangzhou Municipal Science and Technology Special Project for Supporting the Development of Biopharmaceutical and Health Industries (Grants 2023WJC010 and 2024WJC176), the Construction Fund of Key Medical Disciplines of Hangzhou (Grant 2025HZZD19), and the Hangzhou Children’s Hospital Institutional Research Project (Grant ETYY2025Y02).

## Ethics Statement

This study was approved by the Experimental Animal Welfare Ethics Committee of the Zhejiang Academy of Traditional Chinese Medicine (Approval Number 20266, approval date: January 16, 2025). All animal procedures were conducted in accordance with the National Institutes of Health’s Guide for the Care and Use of Laboratory Animals.

## Conflicts of Interest

The authors declare no conflicts of interest.

## Data Availability

The dataset used and/or analyzed during the current study is available from the corresponding author upon reasonable request.
